# Validation of the endothelial staining markers CD31 and CD34 in immunohistochemistry of the long saphenous vein.

**DOI:** 10.1186/1749-8090-10-S1-A321

**Published:** 2015-12-16

**Authors:** B Krishnamoorthy, WR Critchley, JB Barnard, PD Waterworth, AC Caress, J Fildes, N Yonan

**Affiliations:** 1 Department of Cardiothoracic Surgery, University Hospital of South Manchester NHS Foundation Trust, Manchester, M23 9LT, UK; 2 The Transplant Centre, University Hospital of South Manchester NHS Foundation Trust, Manchester, M23 9LT, UK; 3 The School of Nursing, University of Manchester, Manchester, M13 9WL, UK

## Background/Introduction

Endothelial injury during the surgical intervention can significantly affect the functional status of the vein. The endothelial layer plays a vital role in the long saphenous vein for ensuring smooth blood flow and the prevention of vasoconstriction and thrombi formation within the blood vessels. There are few histological studies comparing the different vein harvesting techniques that have studied endothelial layer integrity using CD31 on human long saphenous veins.

## Aims/Objectives

Immunohistochemistry (IHC) remains the gold standard for studying the morphological status of the vein. Although previous studies have used IHC markers to score endothelial integrity, none of them compared the quality of CD34 staining following CABG surgery, particularly in terms of colour, intensity and distribution of their expression. This study aims to compare the endothelial markers CD31 and CD34 as reliable markers of endothelial damage on human long saphenous vein. This is the first study which directly compares these differences to identify and set a standard for future IHC on long saphenous veins.

## Method

Patients were consented prior taking part in this study, which was approved by the Greater Manchester North East - National Research Ethics Committee (NREC). Ten tissue samples were obtained from ten traditional open vein harvesting patients and were automatically processed and stained using immunohistochemistry for CD31 and CD34. The colour, intensity and distribution of the staining on the tissues were scored blindly by five independent scorers and an expert histopathologist for this study. None of these collaborators were involved at any stage of this research project.

## Results

Consecutive saphenous vein sections were stained using anti-CD31 and anti-CD34 antibodies. A significantly different pattern of expression was found in terms of colour, intensity and distribution. The CD34 antibody demonstrated greater colour (p < 0.007), intensity (p < 0.019) and distribution (p < 0.007) compared to CD31.

## Discussion/Conclusion

In conclusion, the use of CD34 for assessing the endothelial integrity is more suitable than CD31. This study provides novel evidence regarding the use of these markers which can have important clinical effects, for example when used for coronary artery bypass surgery.

